# Healthcare setting staff satisfaction in Ethiopia: Systematic review and meta‐analysis

**DOI:** 10.1002/puh2.143

**Published:** 2024-01-10

**Authors:** Gizew Dessie Asres, Yeshiwork Kebede Gessesse

**Affiliations:** ^1^ Amhara Public Health Institute Bahir Dar Amhara Ethiopia; ^2^ Durbete Primary Hospital, Amhara Regional National State Durbete Amhara Ethiopia

**Keywords:** Ethiopia, healthcare settings, healthcare workers, staff satisfaction, systematic review and meta‐analysis

## Abstract

**Background:**

Job satisfaction means how happy and content people are with their job. Workers who are satisfied with their jobs tend to get more work done and give better care. When workers are not happy at work, they are also not productive as well and many of them leave their jobs. This study aimed to address the level of job satisfaction and associated factors among healthcare setting staff in Ethiopia from 2012 to 2022.

**Methods:**

PubMed, Scopus, Cochrane, Google Scholar and African Index Medicus databases have been searched based on preferred reporting items for systematic review and meta‐analysis. Pooled estimate of job satisfaction level was done using the random effects model after significant heterogeneities have been checked by subgroup analysis. Publication bias was checked using funnel plot.

**Results:**

Pooled satisfaction level of healthcare workers in Ethiopia was 50.31%. Factors associated were autonomy (pooled odds ratio (POR) = 5.79, 95% CI: 1.99–16.90), training (POR = 3.09, 95% CI: 1.69–5.67), organizational policy (POR = 4.71, 95% CI: 2.09–10.61), reward (POR = 4.58,95% CI: 1.51–13.84), payment (POR = 3.89, 95% CI: 1.77–8.54), supervision (POR = 5.34, 95% CI: 3.72–7.67) and work environment (POR = 5.44, 95% CI: 2.80–10.58).

**Conclusion:**

About half of healthcare staff in Ethiopia's healthcare settings were satisfied with their job. This result was lower than the job satisfaction level of other parts of the world, even in African countries. Healthcare settings should strive to provide a good working environment, with training opportunities, adequate payment, supportive supervision and conducive organizational policy.

## Graphical abstract

Graphical Abstract: Healthcare setting staff satisfaction in Ethiopia: systematic review and meta‐analysis, 2023.

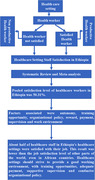

## INTRODUCTION

Job satisfaction or staff satisfaction refers to the attitudes and feelings people have about their work. Positive and favourable attitudes towards jobs indicate staff satisfaction [1]. Staff satisfaction is influenced by a combination of factors, both financial and non‐financial, which contribute to a person's level of contentment with a job [2]. The level of staff satisfaction is affected by intrinsic and extrinsic motivating factors. Researchers identified that the key factors affecting staff satisfaction were career opportunities, job influence, teamwork and job challenge [3, 4].

Staff satisfaction creates loyalty, confidence and commitment to the organization [5]. It leads to improvement and avoids negative behaviour such as absenteeism and turnover [6]. On the other hand, dissatisfied staff are more likely to quit their jobs or be absent than satisfied staff. Job satisfaction shows correlations with turnover and absenteeism. It also appears to be related to other withdrawal behaviours like lateness, unionization, grievances drug abuse, theft or decision to retire [7].

Healthcare setting staff are those who deliver care and services to the sick and ailing either directly or indirectly at health facilities [8, 9]. Healthcare facilities, like other organizations, are concerned with what should be done to achieve sustained high levels of performance through people. This means giving close attention to how healthcare workers can best be motivated through such means as incentives, rewards, leadership and, importantly, the work they do and the organization context within which they carry out that work [1].

Healthcare worker satisfaction influences their productivity, the quality of care they provide, their retention and patient satisfaction [10, 11]. To achieve health‐related sustainable development goals adopted by United Nations in 2015 to reach universal health coverage, adequate, satisfied, competent and engaged health workforce is critically important. Ministries of Health, civil service commissions and employers should adapt employment conditions, remuneration and non‐financial incentives to ensure fair terms for health workers, merit‐based career development opportunities and a positive practice environment to enable their effective deployment, retention and adequate motivation to deliver quality care [12, 13, 14, 15].

So far, staff satisfaction studies among healthcare workers on different parts of the world showed different levels of satisfaction from country to country and discipline to discipline. Staff satisfaction level among United States (US) healthcare workers, Delhi medical officers, French doctors, the Netherlands’ midwives and Iranian nurses was 77.6%, 59.1%, 64%, 100% and 46.3% [16, 17, 18, 19, 20], respectively. Staff satisfaction level among African healthcare workers was 52.1% in South Africa, 71% in Malawi, 82.6% in Tanzania and 62% in Kenya and Ghana [21].

Scholars identified three broad priority areas of concern for all health workers: salary and benefits, opportunities for education, training and career development (promotion) and work environment. Most low‐ and middle‐income countries, including Ethiopia, are constantly looking for strategies to train more health workers and improve job satisfaction, motivation and retention of the health workforce. Although an overwhelming majority of ministries of health and health organizations view staff satisfaction and retention as a key strategic imperative, it is not evident in workforce planning or human resource management operational practice. In other words, although viewed in such a strong light, very few organizations have formal and effectively implemented staff satisfaction and retention strategies [2, 22, 23, 24].

Comprehensive systematic review and meta‐analysis studies on healthcare worker satisfaction in Ethiopia are limited. During our systematic search, we have got three systematic review and meta‐analysis done in Ethiopia. The results are different even though published in the same year. Neither of them clearly stated the condition, context and population (CoCoPop) framework of their respective review and meta‐analysis [25, 26, 27]. Therefore, we conducted this systematic review and meta‐analysis to generate a more comprehensive national healthcare staff satisfaction level using CoCoPop framework.

## METHODS

A systematic review and meta‐analysis following the preferred reporting items for systematic review and meta‐analysis guideline was done to assess the level of healthcare setting staff satisfaction and factors related to it in Ethiopia [28, 29, 30]. A review protocol was developed using Joanna Briggs Institute (JBI) manual for evidence synthesis to predefine objectives and methods of the systematic review [31]. The protocol details the criteria the reviewers have used to include and exclude studies. This protocol was registered from the international prospective register of systematic reviews ID = CRD42022312559.

### Review question

The objective of this systematic review and meta‐analysis was to address the following review questions using the CoCoPop framework:

1. What is the health workforce satisfaction level at healthcare settings in Ethiopia?
2. What are factors for health workforce satisfaction at healthcare setting in Ethiopia?
3. How is the statistical variation of health workforce satisfaction among different disciplines, health facility category (private and public) health facility type (health post, health centre and hospital)?

### Search strategy

We conducted a comprehensive search of PubMed, Scopus, Cochrane, Google Scholar and African Index Medicus databases and grey literatures like WHO library from 16 May 2022 to 30 August 2022. The search terms used at PubMed were ‘health worker’ OR ‘health staff’ OR ‘health care staff’ OR ‘human resource for health’ OR ‘health employee’ OR ‘health professional’ AND ‘health care setting’ OR ‘health facility’ OR ‘health institution’ OR ‘hospital’ OR ‘health centre’ OR ‘clinic’ OR ‘health post’ AND ‘determinants’ OR ‘factors’ OR ‘predictors’ AND ‘satisfaction’ OR ‘job satisfaction’ OR ‘staff satisfaction’ AND ‘Ethiopia’ (Additional file 1).

### Inclusion criteria

The inclusion and exclusion criteria based on the CoCoPop framework were used by reviewers during screening and selection process of articles from bibliographic databases (Table 1).

**TABLE 1:** Inclusion and exclusion criteria for systematic review and meta‐analysis study of healthcare setting staff satisfaction in Ethiopia, 2012–2022.

Included studies	Excluded studies
Job satisfaction studies done on health workers at healthcare settings between 2012 and 2022 in Ethiopia Observational studies with analytical parts Job satisfaction studies done at all levels of healthcare settings Job satisfaction studies on all categories of health workers (clinical and other service providers) Job satisfaction studies done on private or public healthcare settings Studies written in English language Studies having methodological quality assessment >50%	Health workers of Woreda Health Office, Zonal Health Department, Regional Health Bureau, Ministry Off Health, blood bank, Ethiopian Food Drug Administration, professional associations and health science colleges Job satisfaction studies done on health workers at healthcare settings out of Ethiopia Job satisfaction studies done on health workers at healthcare settings before 2012

### Study screening and selection

The process of screening was conducted in two levels. Level one was based on titles and abstracts of the paper. In the second level, papers that have passed from the screening level were downloaded and evaluated against the inclusion criteria. All selected articles from this stage were saved in a separate folder for quality assessment, and those passed the quality assessment were used for this evidence synthesis. Papers that were excluded based on the full‐text assessment against the inclusion and exclusion criteria were justified. The entire screening and selection process was undertaken using Rayyan software [32] independently by two authors (GDA and YKG), who later compared their results from the software. No major discrepancy observed between two authors; however, minor discrepancies observed were resolved based on consensus between the two authors.

### Quality assessment (appraisal) of studies

Critical appraisal tool based on the study design was used to assess the quality of studies. A critical appraisal checklist developed by JBI for prevalence studies has been used for this study (Annex 1). Two reviewers GDA and YKG have appraised the studies independently using the above‐mentioned tool. The tool encompassed nine criteria for rating different quality elements and studies scored 5 and above out of 9 were included in to this meta‐analysis study. During the quality assessment, disagreements were solved through discussion and before agreement reached weighted kappa index was done, which was 98.33% [33, 34].

### Data extraction

JBI standardized data extraction template was used to ensure the extraction of the same types of data across the included studies. The two reviewers GDA and YKG extracted the entire necessary data independently using SRDR+ online platform [35]. The data collection included the following items; study details, study method and results.

### Data synthesis and meta‐analysis

The data synthesized within this systematic review were the results extracted from individual research studies relevant to the review question. Narrative summary in this systematic review utilized tables, graphs and pictorial diagrams to help convey how studies compare to each other and to assist in the presentation of the data. It has included the presentation of the quantitative results reported in individual studies, the point estimates and the interval estimate at 95% confidence intervals for level of staff satisfaction and associated factors.

Meta‐analysis was conducted to figure out the pool health worker satisfaction level using random and fixed effect models. All the included studies were synthesized according to outcome variables and study designs using Meta essentials v 1.5 [36, 37] and Med Calc v 20.114 [38] statistical tools. The extent of heterogeneity across studies was checked using *Q*‐test and *I* ^2^‐test (*I* ^2^ > 50% indicating significant heterogeneity). Subgroup analysis was conducted on the sample size of individual studies, year of publication, healthcare setting, region and health worker categories. To study the effect of covariates on the pooled effect size and the heterogeneity across studies, subgroup analysis was performed. The effect of covariates on the pooled effect size was considered significant when the *p*‐value is <0.05 or their 95% CI were not crossed one.

## RESULTS

### Screening and selection process of included studies

**FIGURE 1:**
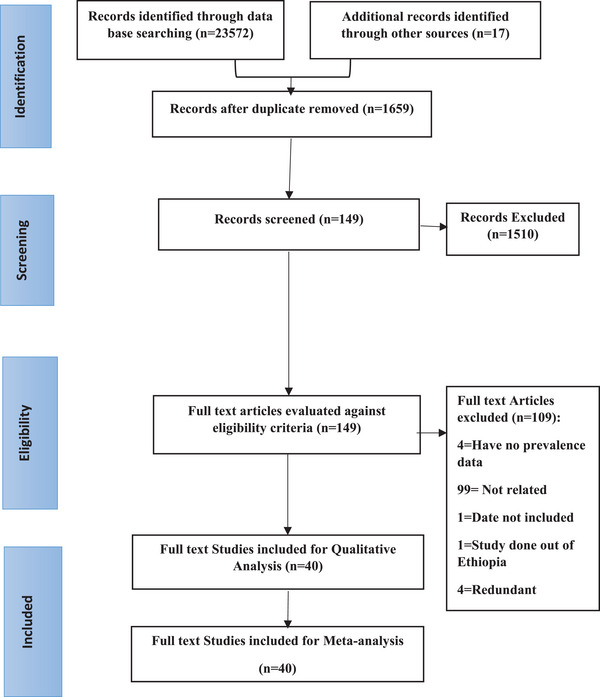
Flowchart presenting the study selection with the preferred reporting items for systematic reviews and meta‐analyses (PRISMA) guidelines for systematic review and meta‐analysis on healthcare setting staff satisfaction, 2023.

During bibliographic database search, a total of 23,589 studies were identified of which 21,930 were duplicates and removed. Of 1659 remaining studies, 1510 were found to be unrelated and excluded based on title and abstract screening. Based on eligibility criteria, 109 full‐text articles were not eligible and removed. Four full‐text articles excluded due to the unavailability of the overall prevalence of job satisfaction data. Ninety‐nine full‐text articles found unrelated for this systematic review. Four full‐text articles were redundant, one full‐text article was out of Ethiopia, and one full‐text article was out of recommended date and excluded. Finally, we end up with 40 full‐text articles [39, 40, 41, 42, 43, 44, 45, 46, 47, 48, 49, 50, 51, 52, 53, 54, 55, 56, 57, 58, 59, 60, 61, 62, 63, 64, 65, 66, 67, 68, 69, 70, 71, 72, 73, 74, 75, 76, 77] for systematic review and meta‐analysis (Figure 1).

### Characteristics of included studies

Among 40 included studies published from 2012 to 2022 in Ethiopia, the smallest sample size was 60 on a study done in Tigray regional state, and the largest one was 575 on a study done in Amhara regional state. The study settings included were health posts, health centres, hospitals, community pharmacies, private hospitals and clinics. These studies involved a total of 10,749 participants from health extension workers, nurses, midwives, pharmacy professionals, anaesthetists and doctors (Table 2).

**TABLE 2:** Characteristics of included studies for systematic review and meta‐analysis of healthcare setting staff satisfaction in Ethiopia (*n* = 40), 2023.

First author, reference	Region	Study setting	Participants	Study design	Sampling procedure	Sample size	Event	Prevalence (%)
Admasu [39]	Oromia	Hospital	Nurses	CS	Simple RS	98	47	48
Agezegn Asegid [40]	SNNP	HC and hospital	Nurses	CS	Simple RS	242	127	52.5
Amare Geta [41]	Amhara	Hospital	All	CS	Stratified simple RS	259	75	29
Amare Geta [41]	Amhara	Private hospital	All	CS	Stratified simple RS	261	212	81.2
ASCHALEW MESFIN (2022) [42]	Amhara	Hospital	All	CS	Simple RS	242	117	48.3
Assefa Philipos Kare [43]	Sidama	Health post	HEW	CS	Simple RS	339	124	36.6
Ayele Geleto [44]	Harari	HC and hospital	All	CS	Simple RS	405	179	44.2
Ayele Semachew [45]	Oromia	Hospital	Nurses	CS	Census	316	213	67.4
Belsity Temesgen Meselu [46]	Tigray	HC	Midwives	CS	Simple RS	140	61	43.6
Beyazin Kebede Deriba [47]	Oromia	HC	All	CS	Simple RS	308	128	41.5
David R. Hotchkiss [48]	AA, Oromia, Amhara and Somalia	HC, clinic, health post	All	CS	Purposive sampling	312	248	79.5
Dawit Gebregziabher [49]	Tigray	Hospital	Nurses	CS	Systematic RS	148	130	87.60
Demeke Yilkal Fentie [50]	Amhara	Hospital	Anaesthetists	CS	purposive sampling	98	46	46.90
Emiru Ayalew [51]	Amhara	HC and hospital	Nurses	CS	Simple RS	220	96	43.60
Endager Abera [52]	Amhara	Hospital	All	CS	Simple RS	394	191	48.50
Eyasu Tamru Bekru [53]	AA	HC and hospital	Midwives	CS	Simple RS	221	117	52.90
Firew Ayalew [54]	Ethiopia	HC and hospital	Nurses	CS	Simple RS	390	237	60.80
Getnet Gedif (2018)	Amhara	Hospital	All	CS	Simple RS	383	207	54.00
Hailu Merga [55]	Oromia	HC and hospital	All	CS	Systematic RS	415	160	38.50
Kalkidan Temesgen [56]	Amhara	HC and hospital	All	CS	Simple RS	575	182	31.70
Mesfin Aklilu [57]	AA	HC	All	CS	Simple RS	379	204	53.80
Mulugeta Mekuria Mengistu [58]	Oromia	HC and hospital	All	CS	Simple RS	166	58	34.90
Muluken Dessalegn Muluneh [59]	Ethiopia	HC and hospital	Midwives	CS	Simple RS	107	45	42.00
Nebiat Negussie [60]	Ethiopia	Hospital	Nurses	CS	Simple RS	327	277	84.57
Nega Desalegn [61]	Ethiopia	Hospital	Anaesthetists	CS	Simple RS	242	131.164	54.20
Seid Mussa Ahmed [62]	Oromia	HC and hospital	Pharmacists	CS	Purposive sampling	97	59	60.80
Sharon Kibwana [63]	Ethiopia	Hospital	Anaesthetists	CS	Simple RS	252	107	42.50
Tirhas Tadese [64]	Addis Ababa	Hospital	All	CS	purposive sampling	296	125	42.20
Tsegahun Manyazewa [65]	Addis Ababa	Hospital	All	CS	Purposive sampling	410	201	49.00
Wondwossen Yimam [66]	Addis Ababa	Hospitals	All	CS	Systematic RS	314	116	37.00
Yohanes Ayele [67]	Oromia, Somalia	Hospitals	Pharmacy	CS	Simple RS	220	72	32.70
Ebrahim Mohammed [68]	Oromia	Hospital	All	CS	Stratified RS	264	113	42.80
Abera Merga [69]	AA	Hospital	Nurses	CS	Simple RS	135	45	33.30
Azagew and Mekonen [77]	Amhara	Hospital	Nurses	CS	Simple RS	406	203	50.00
Tegbar_Yigzaw_ [70]	Ethiopia	Hospital	GP	CS	Simple RS	502	221	39.20
Mohammed et al. [72]	Harari	Community pharmacy	Pharmacy prof	CS	Simple RS	73	40	54.80
Muktar Abadiga [73]	Oromia	Hospital	Nurses	CS	Simple RS	252	130	51.60
Belay and Practice [74]	Tigray	Community pharmacy	Pharmacy pro	CS	Purposive sampling	60	41	68.30
Desalegn Haile [75]	Amhara	Hospitals	Nurses	CS	Simple RS	176	96	54.50
Addisu Abebe Teka (2018)	Oromia	HC and hospitals	All	CS	Simple RS	305	105	34.40

Abbreviations: All, when participants are more than one health professions; CS, cross‐sectional study; HC, health centre; HEW, health extension worker; RS, random sampling.

### Methodological quality of included studies

Methodological quality assessment was done using the JBI‐Prevalence Critical Appraisal Checklist. Assessment was done by two independent assessors, GDA and YKG. Discrepancies between assessors were resolved through consensus and mutual understanding. The assessment results revealed that the quality of included studies ranges from 77.8% to 100% (Table 3).

**TABLE 3:** Methodological quality assessment results of included studies for systematic review and meta‐analysis of healthcare setting staff satisfaction in Ethiopia (*n* = 40), 2023.

Study code	Was the sample frame appropriate to address the target population? (1)	Were study participants sampled in an appropriate way? (2)	Was the sample size adequate? (3)	Were the study subjects and the setting described in detail? (4)	Was the data analysis conducted with sufficient coverage of the identified sample? (5)	Were valid methods used for the identification of the condition? (6)	Was the condition measured in a standard, reliable way for all participants? (7)	Was there appropriate statistical analysis? (8)	Was the response rate adequate, and if not, was the low response rate managed appropriately? (9)	Total yes/comment (%)
Admasu BG [39]	Yes	Yes	No	Yes	Yes	Yes	Yes	Yes	Yes	88.9
Agezegn Asegid [40]	Yes	Yes	Yes	Yes	Yes	Yes	Yes	Yes	No	88.9
Amare Geta [41]	Yes	Yes	Yes	Yes	Yes	Yes	Yes	Yes	Yes	100
Amare Geta [41]	Yes	Yes	Yes	Yes	Yes	Yes	Yes	Yes	Yes	100
ASCHALEW MESFIN (2022) [42]	Yes	Yes	Yes	Yes	Yes	Yes	Yes	Yes	Yes	100
Assefa Philipos Kare [43]	Yes	Yes	Yes	Yes	Yes	Yes	Yes	Yes	Yes	100
Ayele Geleto [44]	Yes	Yes	Yes	Yes	Yes	Yes	Yes	Yes	Yes	100
Ayele Semachew [45]	Yes	Yes	Yes	Yes	Yes	Yes	Yes	Yes	Yes	100
Belsity Temesgen Meselu [46]	Yes	Yes	No	Yes	Yes	Yes	Yes	Yes	Yes	88.9
Beyazin Kebede Deriba [47]	Yes	Yes	Yes	Yes	Yes	Yes	Yes	Yes	Yes	100
David R. Hotchkiss [48]	Yes	No	Yes	Yes	Yes	Yes	Yes	Yes	Yes	88.9
Dawit Gebregziabher [49]	Yes	Yes	No	Yes	Yes	Yes	Yes	Yes	Yes	88.9
Demeke Yilkal Fentie [50]	Yes	No	No	Yes	Yes	Yes	Yes	Yes	Yes	77.8
Emiru Ayalew [51]	Yes	Yes	Yes	Yes	Yes	Yes	Yes	Yes	Yes	100
Endager Abera [52]	Yes	Yes	Yes	Yes	Yes	Yes	Yes	Yes	Yes	100
Eyasu Tamru Bekru [53]	Yes	Yes	Yes	Yes	Yes	Yes	Yes	Yes	Yes	100
Firew Ayalew [54]	Yes	Yes	Yes	Yes	Yes	Yes	Yes	Yes	No	88.9
Getnet Gedif (2018)	Yes	Yes	Yes	Yes	Yes	Yes	Yes	Yes	Yes	100
Hailu Merga [55]	Yes	Yes	Yes	Yes	Yes	Yes	Yes	Yes	Yes	100
Kalkidan Temesgen [56]	Yes	Yes	Yes	Yes	Yes	Yes	Yes	Yes	No	88.9
Mesfin Aklilu [57]	Yes	Yes	Yes	Yes	Yes	Yes	Yes	Yes	Yes	100
Mulugeta Mekuria Mengistu [58]	Yes	Yes	No	Yes	Yes	Yes	Yes	Yes	Yes	88.9
Muluken Dessalegn Muluneh [59]	Yes	Yes	No	Yes	Yes	Yes	Yes	Yes	Yes	88.9
Nebiat Negussie [60]	Yes	Yes	Yes	Yes	Yes	Yes	Yes	Yes	Yes	100
Nega Desalegn [61]	Yes	Yes	Yes	Yes	Yes	Yes	Yes	Yes	Yes	100
Seid Mussa Ahmed [62]	Yes	No	No	Yes	Yes	Yes	Yes	Yes	Yes	77.8
Sharon Kibwana [63]	Yes	Yes	Yes	Yes	Yes	Yes	Yes	Yes	Yes	100
Tirhas Tadese [64]	Yes	No	Yes	Yes	Yes	Yes	Yes	Yes	Yes	88.9
Tsegahun Manyazewa [65]	Yes	No	Yes	Yes	Yes	Yes	Yes	Yes	No	77.8
Wondwossen Yimam [66]	Yes	Yes	Yes	Yes	Yes	Yes	Yes	Yes	Yes	100
Yohanes Ayele [67]	Yes	Yes	Yes	Yes	Yes	Yes	Yes	Yes	Yes	100
Ebrahim Mohammed [68]	Yes	Yes	Yes	Yes	Yes	Yes	Yes	Yes	Yes	100
Abera Merga [69]	Yes	Yes	Yes	Yes	Yes	Yes	Yes	Yes	Yes	100
Azagew and Mekonen [77]	Yes	Yes	Yes	Yes	Yes	Yes	Yes	Yes	Yes	100
Sendekie et al. (2020)	Yes	Yes	Yes	Yes	Yes	Yes	Yes	Yes	Yes	100
Mohammed et al. [72]	Yes	Yes	No	Yes	Yes	Yes	Yes	Yes	Yes	88.9
Muktar Abadiga [73]	Yes	Yes	Yes	Yes	Yes	Yes	Yes	Yes	Yes	100
Belay and Practice [74]	Yes	Yes	Yes	Yes	Yes	Yes	Yes	Yes	Yes	100
Desalegn Haile [75]	Yes	Yes	No	Yes	Yes	Yes	Yes	Yes	Yes	88.9
Addisu Abebe Teka (2018)	Yes	Yes	No	Yes	Yes	Yes	Yes	Yes	Yes	88.9

### Findings of the review

**FIGURE 2:**
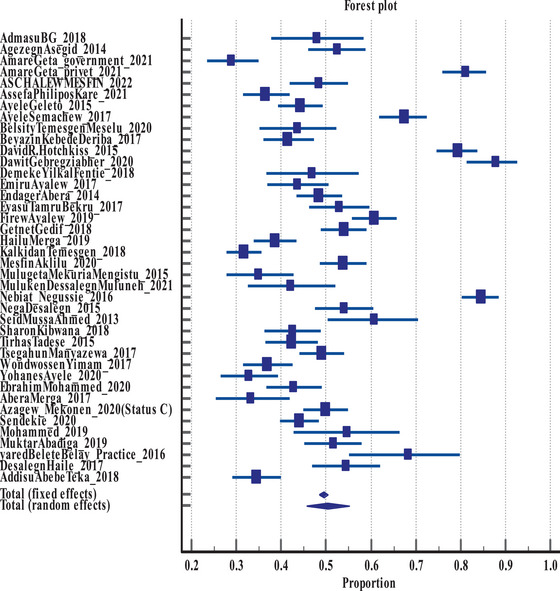
Forest plot showing the effect size of staff satisfaction.

Based on 40 included studies with 10,749 participants, the pooled satisfaction level of health workers at Ethiopian healthcare setting was found to be 49.41% (95%, CI: 48.47–50.36) for fixed effect model and 50.31%(95%, CI: 45.59–55.01) for Random effect model at Q = Q = 957.8331 (*I* ^2^ (inconsistency) = 95.93%, *p* < 0.0001). Only the result from random effect model was used for further discussion as there was significant heterogeneity between studies (Table 4 and Figure 2).

**TABLE 4:** Level of healthcare worker satisfaction at healthcare settings in Ethiopia, 2023.

				Weight (%)	
Study ID	Sample size	Proportion (%)	95% CI	Fixed	Random
Admasu BG_2018	98	47.96	37.76–58.29	0.92	2.36
Agezegn Asegid_2014	242	52.48	45.98–58.91	2.25	2.52
Amare Geta_government_2021	259	28.96	23.51–34.89	2.41	2.53
AmareGeta_privet_2021	261	81.23	75.95–85.78	2.43	2.53
ASCHALEW MESFIN_2022	242	48.35	41.90–54.84	2.25	2.52
Assefa Philipos Kare_2021	339	36.58	31.44–41.95	3.15	2.55
Ayele Geleto_2015	405	44.20	39.30–49.19	3.76	2.57
Ayele Semachew_2017	316	67.41	61.93–72.55	2.94	2.55
Belsity Temesgen Meselu_2020	140	43.57	35.22–52.20	1.31	2.44
Beyazin Kebede Deriba_2017	308	41.56	35.99–47.28	2.86	2.54
DavidR.Hotchkiss_2015	312	79.49	74.58–83.83	2.90	2.54
Dawit Gebregziabher_2020	148	87.84	81.46–92.63	1.38	2.45
Demeke YilkalFentie_2018	98	46.94	36.78–57.29	0.92	2.36
Emiru Ayalew_2017	220	43.64	36.98–50.47	2.05	2.51
Endager Abera_2014	394	48.48	43.44–53.53	3.66	2.56
Eyasu Tamru Bekru_2017	221	52.94	46.13–59.67	2.06	2.51
Firew Ayalew_2019	390	60.77	55.73–65.65	3.62	2.56
Getnet Gedif_2018	383	54.05	48.91–59.12	3.56	2.56
HailuMerga_2019	415	38.55	33.85–43.43	3.86	2.57
Kalkidan Temesgen_2018	575	31.65	27.87–35.63	5.34	2.59
Mesfin Aklilu_2020	379	53.83	48.67–58.93	3.52	2.56
Mulugeta Mekuria Mengistu_2015	166	34.94	27.72–42.71	1.55	2.47
Muluken Dessalegn Muluneh_2021	107	42.06	32.58–51.99	1.00	2.39
Nebiat_Negussie_2016	327	84.71	80.34–88.43	3.04	2.55
Nega Desalegn_2015	242	54.13	47.63–60.53	2.25	2.52
Seid Mussa Ahmed_2013	97	60.83	50.39–70.58	0.91	2.36
Sharon Kibwana_2018	252	42.46	36.28–48.82	2.34	2.52
Tirhas Tadese_2015	296	42.23	36.54–48.08	2.75	2.54
Tsegahun Manyazewa_2017	410	49.02	44.09–53.98	3.81	2.57
Wondwossen Yimam_2017	314	36.94	31.59–42.54	2.92	2.55
Yohanes Ayele_2020	220	32.73	26.57–39.36	2.05	2.51
Ebrahim Mohammed_2020	264	42.80	36.77–49.01	2.46	2.53
Abera Merga_2017	135	33.33	25.46–41.96	1.26	2.43
Azagew_Mekonen_2020 (Status C)	406	50.00	45.03–54.97	3.77	2.57
Sendekie_2020	502	44.02	39.63–48.49	4.66	2.58
Mohammed_2019	73	54.80	42.71–66.48	0.69	2.29
MuktarAbadiga_2019	252	51.59	45.23–57.91	2.34	2.52
Yared Belete Belay_Practice_2016	60	68.33	55.04–79.74	0.57	2.22
Desalegn Haile_2017	176	54.55	46.88–62.05	1.64	2.48
Addisu Abebe Teka_2018	305	34.43	29.10–40.05	2.84	2.54
Total (fixed effects)	10749	**49.41**	**48.47–50.36**	100.00	100.00
Total (random effects)	10749	**50.31**	**45.59–55.01**	100.00	100.00
Heterogeneity	*I* ^2^ (inconsistency) = 95.93%, CI: 95.13–96.60, *p* < 0.001, *Q* = 957.8331				

### Publication bias analysis of included studies

**FIGURE 3:**
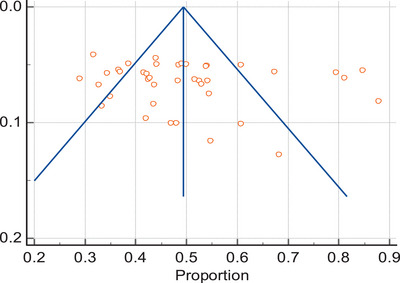
Funnel plot showing symmetry of included articles.

Egger's test and symmetric funnel plot showed that studies included in this meta‐analysis have no publication bias (Figure 3).

### Sub group analysis

To investigate the source of heterogeneity, sub group analysis has been done on sample size, study area, participant's profession, year of publication and health facility type. All the above listed variables were significant source of statistical heterogeneity of which health worker category was the highest source of heterogeneity (*I* ^2^ = 99.48%, Table 5).

**TABLE 5:** Sub group analysis on the level of staff satisfaction using meta essentials.

						Weight (%)		
Variable	Characteristics	Sample size	Event	Odds ratio	95% CI	Fixed	Random	*p*‐Value
Sample size	<100	426	233	1.35	1.11–1.64	0.75	3.84	0.621
*Q* = 83.72	100–199	872	435	1.11	0.97–1.28	1.53	4.04	
*p* < 0.001	200–300	3791	1619	0.76	0.70–0.82	6.66	4.21	
*I* ^2^ = 96.42%	>300	6886	3395	1.19	1.11–1.28	12.09	4.24	
Study area/region	AA	2461	1118	1.01	0.92–1.11	4.32	4.18	0.692
*I* ^2^ = 96.45%	Amhara	3014	1425	0.30	0.27–0.34	5.29	4.20	
*p* < 0.001	Harari	478	219	1.02	0.85–1.23	0.84	3.88	
*Q* = 112.69	Oromia	2741	1099	0.74	0.68–0.81	4.81	4.19	
	Tigray	348	232	2.50	1.99–3.14	0.61	3.75	
Participants/health worker category	Anaesthesia	592	284	1.02	0.87–1.21	1.04	3.95	0.337
*I* ^2^ = 99.48%	HEW	859	210	0.07	0.06–0.08	1.51	4.04	
*p* < 0.001	Midwives	468	223	1.01	0.84–1.21	0.82	3.87	
*Q* = 1145.61	Nurses	2710	1601	1.83	1.68–2.00	4.76	4.19	
	Pharmacy	450	212	0.99	0.82–1.19	0.79	3.86	
	All	6394	2931	0.87	0.81–0.94	11.23	4.23	
	GP	502	221	0.87	0.72–1.04	0.88	3.90	
Publication year	2013–2017	5347	2758	1.35	1.26–1.45	9.39	4.23	1.000
*I* ^2^ = 99.24% *Q* = 132.01 *p* < 0.001	2018–2022	6628	2924	0.74	0.69–0.80	11.64	4.24	
Study setting/type of health facility	HC	827	393	1.00	0.87–1.16	1.45	4.03	0.142
*Q* = 447.6679	Hospital	6440	3173	1.17	1.09–1.26	11.31	4.23	
*I* ^2^ = 98.66%	HC and hospital	3143	1365	0.57	0.52–0.63	5.52	4.20	
*p* < 0.001	Community pharmacy	133	81	1.74	1.22–2.46	0.24	3.14	
	HC, clinic, health post	312	248	4.44	3.37–5.86	0.55	3.70	
	Health post	859	210	0.33	0.28–0.39	1.51	4.04	
	Private hospital	261	212	4.94	3.61–6.75	0.46	3.61	

### Factors associated with health worker's job satisfaction at healthcare settings in Ethiopia

Among 40 included studies used for pooled satisfaction level analysis, 15 were assessed for factors associated with job satisfaction. Factors assessed were as follows: having additional responsibility, job autonomy, job description, co‐worker relationship, further educational opportunity, management support, nature of work, on‐job training, organizational policy, performance appraisal, reward/recognition, satisfied with monthly salary, supervision, work achievement, work environment, working equipment availability and workload. Of which autonomy (pooled odds ratio (POR) = 5.79, 95% CI: 1.99–16.90), on‐job training (POR = 3.09, 95% CI: 1.69–5.67), organizational policy (POR = 4.71, 95% CI: 2.09–10.61), reward/recognition (POR = 4.58, 95% CI: 1.51–13.84), satisfied with monthly salary (POR = 3.89, 95% CI: 1.77–8.54), adequate supervision (OR = 5.34, 95% CI: 3.72–7.67) and work environment (POR = 5.44, 95% CI: 2.80–10.58) were found to be significantly associated factors for overall staff satisfaction of health workers at healthcare setting in Ethiopia (Table 6).

**TABLE 6:** Factors associated with healthcare workers’ job satisfaction at healthcare settings in Ethiopia, 2023.

Factors	Study ID	Odds ratio	CI	Weight (%)	Pooled odds ratio	*Q*	*I* ^2^ (%)	*T* ^2^
**Additional responsibility (head or coordinator)**	Amare Geta [41] private	5.40	2.78–10.46	**4.81**	**3.81 (0.95**–**15.24)**	**20.61**	**85.44**	**0.61**
	Amare Geta [41] public	2.96	1.58–5.55					
	Eyasu Tamru Bekru [53]	1.21	0.54–2.71					
	Tirhas Tadese [64]	9.46	5.70–15.70					
**Autonomy**	Amare Geta [41] private	6.99	3.55–13.75	**8.49**	**5.79 (1.99**–16.90)*	**4.64**	**56.92**	**0.11**
	Amare Geta [41] public	3.49	1.95–6.28					
	Ebrahim mohammed [68]	7.67	4.88–12.07					
**Clear job description**	Amare Geta [41] private	5.78	2.20–15.23	**4.89**	**2.89 (0.45**–**18.40)**	**6.22**	**67.85**	**0.39**
	Amare Getat [41] public	3.53	1.51–8.23					
	Mulugeta Mekuria Mengistu [58]	1.35	0.64–2.82					
**Co‐worker relationship**	Amare Geta [41] private	3.11	1.40–6.94	**4.43**	**1.92 (0.58**–**6.33)**	**41.09**	**87.83**	**0.87**
	Amare Geta [41] public	1.57	0.86–2.88					
	Aynye Negesse Woldekiro (2022)	0.23	0.08–0.65					
	Ebrahim mohammed [68]	5.53	3.57–8.55					
	Emiru Ayalew [51]	4.68	2.05–10.67					
	Tirhas Tadese [64]	1.27	0.62–2.62					
**Further education opportunity**	Assefa Philipos Kare [43]	3.25	1.98–5.33	**5.40**	**1.91 (0.64**–**5.76)**	**50.85**	**92.13**	**0.80**
	Amare Geta [41] public	2.05	1.02–4.12					
	Sharon Kibwana [63]	0.94	0.60–1.48					
	Tirhas Tadese [64]	0.69	0.41–1.16					
	Tsegahun Manyazewa [65]	5.93	3.69–9.54					
**Management support**	Assefa Philipos Kare [43]	4.35	2.71–6.99	**1.61**	**1.13 (0.07**–**18.62)**	**88.85**	**96.62**	**2.16**
	Aynye Negesse Woldekiro (2022)	0.64	0.38–1.08					
	Belsity Temesgen Meselu [46]	0.11	0.05–0.26					
	Tsegahun Manyazewa [65]	4.61	2.90–7.34					
**Nature of work**	Amare Geta [41] private	0.13	0.07–0.26	**2.67**	**0.84 (0.14**–**5.15)**	**74.14**	**94.60**	**1.59**
	Amare Geta [41] public	0.33	0.19–0.57					
	Emiru Ayalew [51]	5.70	2.61–12.45					
	Eyasu Tamru Bekru [53]	0.91	0.52–1.58					
	Getnet Gedif (2018)	1.93	1.20–3.08					
**On‐job training**	Amare Geta [41] private	2.11	1.11–4.01	**9.28**	**3.09 (1.69**–5.67)*	**11.50**	**65.21**	**0.17**
	Amare Geta [41] public	1.81	1.01–3.25					
	Assefa Philipos Kare [43]	5.87	3.55–9.70					
	Aynye Negesse Woldekiro (2022)	3.94	2.20–7.07					
	Belsity Temesgen Meselu [46]	2.91	1.44–5.86					
**Organizational policy**	Amare Geta [41] private	3.69	1.90–7.16	**10.07**	**4.71 (2.09**–10.61)*	**2.09**	**4.47**	**0.00**
	Amare Geta [41] public	4.01	2.13–7.53					
	Emiru Ayalew [51]	6.68	3.67–12.16					
**Performance appraisal**	Amare Geta [41] private	0.13	0.07–0.26	**3.91**	**1.36 (0.41**–**4.51)**	**162.56**	**95.69**	**2.05**
	Amare Geta [41] public	0.33	0.19–0.57					
	Emiru Ayalew [51]	5.70	2.61–12.45					
	Eyasu Tamru Bekru [53]	0.91	0.52–1.58					
	Getnet Gedif (2018)	1.93	1.20–3.08					
	Amare Geta [41] private	1.40	0.58–3.35					
	Amare Geta [41] public	1.66	0.88–3.10					
	Ebrahim mohammed [68]	11.47	7.12‐ 18.47					
**Reward and recognition**	Amare Geta [41] private	1.75	0.77–3.98	**5.38**	**4.58 (1.51**–13.84)*	**43.40**	**90.78**	**0.88**
	Amare Geta [41] public	4.86	2.21–10.68					
	Ebrahim mohammed [68]	13.97	8.56–22.80					
	Emiru Ayalew [51]	7.88	4.18–14.86					
	Getnet Gedif (2018)	2.02	1.34–3.06					
**Satisfied with monthly salary**	Eyasu Tamru Bekru [53]	5.49	3.06–9.85	**6.80**	**3.89 (1.77**–8.54)*	**37.52**	**84.01**	**0.51**
	Assefa Philipos Kare [43]	5.56	3.41–9.07					
	Aynye Negesse Woldekiro (2022)	3.10	1.75–5.51					
	Belsity Temesgen Meselu [46]	6.67	2.08–21.40					
	Getnet Gedif (2018)	2.44	1.40–4.26					
	Muluken Dessalegn Muluneh [59]	0.76	0.34–1.69					
	Tsegahun Manyazewa [65]	10.78	6.34–18.30					
**Supervision**	Amare Geta [41] private	7.18	3.55–14.53	**11.15**	**5.34 (3.72**–7.67)*	**14.90**	**59.73**	**0.11**
	Amare Geta [41] public	4.80	2.70–8.54					
	Assefa Philipos Kare [43]	8.21	4.94–13.63					
	Ebrahim mohammed [68]	6.66	4.24–10.46					
	Emiru Ayalew [51]	5.01	2.80‐ 8.96					
	Eyasu Tamru Bekru [53]	5.64	3.15–10.12					
	Getnet Gedif (2018)	2.65	1.73–4.06					
**Work achievement**	Emiru Ayalew [51]	5.58	2.89–10.76	**2.95**	**1.96 (0.14**–**27.44)**	**25.16**	**92.05**	**0.85**
	Eyasu Tamru Bekru [53]	0.66	0.38–1.14					
	Getnet Gedif (2018)	2.13	1.40–3.23					
**Work environment**	Amare Geta [41] private	4.77	2.46–9.27	**7.70**	**5.44 (2.80**–10.58)*	**54.44**	**87.14**	**0.51**
	Amare Geta [41] public	2.24	1.29–3.88					
	Ayele Geleto [44]	3.36	2.23–5.07					
	Belsity Temesgen Meselu [46]	15.25	6.43–36.19					
	Ebrahim mohammed [68]	11.47	7.12–18.47					
	Emiru Ayalew [51]	13.07	6.33–26.96					
	Getnet Gedif (2018)	1.94	1.24–3.06					
	Tsegahun Manyazewa [65]	6.41	3.95 −10.41					
**Working equipment available**	Ayele Geleto [44]	2.17	1.44–3.25	**9.09**	**2.52 (0.14**–**44.07)**	**1.34**	**25.43**	**0.03**
	Belsity Temesgen Meselu [46]	3.54	1.71–7.33					
**Workload**	Eyasu Tamru Bekru [53]	8.22	4.48–15.08	**1.35**	**2.33 (0.11**–**51.87)**	**167.05**	**98.20**	**3.37**
	Kalkidan Temesgen [56]	4.75	3.25 −6.95					
	Tirhas Tadese [64]	0.13	0.08–0.21					
	Tsegahun Manyazewa [65]	5.96	3.64–9.76					

* *p* < 0.05.

## DISCUSSION

From this review, we generalized that the pooled job satisfaction level at healthcare settings in Ethiopia was 50.31% at 95% CI (45.59%–55.01%). This result showed that job satisfaction level among healthcare setting staff in Ethiopia was lower than job satisfaction level among US healthcare workers (77.6%), Delhi medical officers (59.1%), French doctors (64%) and the Netherlands’ midwives (100%). It is also lower than job satisfaction level among African healthcare workers; 52.1% in South Africa, 71% in Malawi, 82.6% in Tanzania and 62% in Kenya and Ghana [21]. On the other hand, job satisfaction level in Ethiopia healthcare settings was higher than Iranian nurses (46.3%) [16, 17, 18, 19, 20]. The above‐mentioned differences in the level of satisfaction among healthcare setting workers may be variations in work autonomy, training opportunity, organizational policy, reward/recognition, payment scheme, adequate supervision and working environment.

Significant heterogeneity was observed in regional states on the satisfaction level as 66.67% in Tigray regional state, 47.28% in Amhara regional state, 45.82% in Harari regional state, 45.43% in Addis Ababa city administration and 40.09% in Oromia regional state. Staff satisfaction level based on healthcare settings was 81.23%, 60.90%, 49.27%, 47.52% and 24.45% at private hospitals, community pharmacies, government hospitals, health centres and health posts, respectively. Based on health worker categories, the satisfaction level was 59.08%, 47.97%, 47.65%, 47.11%, 44.02% and 24.45% among nurses, anaesthetists, midwives, pharmacy professionals, doctors and HEWs, respectively.

Factors associated with staff satisfaction at this systematic review were as follows: on‐job training, organizational policy, reward/recognition, job autonomy, monthly salary, supervision and work environment. Health workers who had job autonomy were 5.79 times more likely to be satisfied with their job than who had no job autonomy. Those health workers who thought their work environment was conducive were 5.44 times more likely to be satisfied with their job than who did not think their work environment was conducive. Health workers who got adequate supervision were 5.34 times more likely to be satisfied with their job than who did not get adequate supervision. Those health workers who thought of suitable organizational policy were 4.71 times more likely to be satisfied with their job than who did not think that their organizational policy was suitable. Those who thought of fair reward and recognition at their organization were 4.58 times more likely to be satisfied with their job than who did not think of fair reward and recognition at their organization. Those healthcare workers who satisfied with their salary were 3.89 times more likely to be satisfied with their job than who did not satisfied with their monthly salary. Those who got on‐job training were 3.09 times more likely to be satisfied than who did not get on‐job training.

### Limitation

This systematic review and meta‐analysis have some limitations. First, included studies were heterogeneous with respect to study procedures, participants and study settings. Accordingly, our findings were summarized on a broader level, which inherently suppresses some of the unique features of different approaches. Second, the majority of studies were based on cross‐sectional study design that limits the precision of job satisfaction results. Third, the included articles were only English language studies with full‐text articles freely accessible that compromises the quality and generalizability of the review.

## CONCLUSION

About half of the healthcare workers in healthcare setting in Ethiopia were satisfied with their job. This result was lower than the job satisfaction level of other parts of the world, even the African countries. This discrepancy might be due to variations in socio‐economic, political and studied health worker categories. Associated factors with job satisfaction by this systematic review were as follows: on‐the‐job training, organizational policy, reward/recognition, job autonomy, monthly salary, supervision and work environment.

## RECOMMENDATION

Healthcare settings should strive to provide a good working environment for employees, with opportunities for training, adequate monthly salary supportive supervision and conducive organizational policy. To be more specific and precise, the authors recommend further systematic review and meta‐analysis on a departmental basis and conduct an original study on administrative staff who are working in finance, human resource and health professionals who are small in number, like environmental health.

## AUTHOR CONTRIBUTIONS

Gizew Dessie Asres and Yeshiwork Kebede Gessesse contributed to the conception and design of the review. Gizew Dessie Asres and Yeshiwork Kebede Gessesse were responsible for screening, data extraction, synthesis and analysis of the data. Gizew Dessie Asres drafted the manuscript, which was critically revised by Yeshiwork Kebede Gessesse. Rechecking together was done before the final stage of the manuscript.

## CONFLICT OF INTEREST STATEMENT

The authors have no conflicts of interest.

## ETHICS STATEMENT

An ethical statement is not applicable as this publication did not involve human or animal research as it is a systematic review paper.

## Data Availability

All necessary information was attached to this manuscript. Whenever an additional data set is needed, data sharing applies upon reasonable request to the corresponding author.
